# Neurofilament light chain for classifying the aetiology of alteration of consciousness

**DOI:** 10.1093/braincomms/fcad278

**Published:** 2023-10-18

**Authors:** Tatchaporn Ongphichetmetha, Poosanu Thanapornsangsuth, Watayuth Luechaipanit, Nattawan Loymunkong, Wanakorn Rattanawong, Akarin Hiransuthikul, Thirawat Supharatpariyakorn, Sira Sriswasdi, Thiravat Hemachudha

**Affiliations:** 1 Division of Neurology, Department of Medicine, Faculty of Medicine, Chulalongkorn University, Bangkok 10330, Thailand; Siriraj Neuroimmunology Center, Department of Medicine, Faculty of Medicine Siriraj Hospital, Mahidol University, Bangkok 10700, Thailand; 1 Division of Neurology, Department of Medicine, Faculty of Medicine, Chulalongkorn University, Bangkok 10330, Thailand; Thai Red Cross Emerging Infectious Diseases Health Science Centre, World Health Organization Collaborating Centre for Research and Training on Viral Zoonoses, King Chulalongkorn Memorial Hospital The Thai Red Cross Society, Bangkok 10330, Thailand; Thai Red Cross Emerging Infectious Diseases Health Science Centre, World Health Organization Collaborating Centre for Research and Training on Viral Zoonoses, King Chulalongkorn Memorial Hospital The Thai Red Cross Society, Bangkok 10330, Thailand; Thai Red Cross Emerging Infectious Diseases Health Science Centre, World Health Organization Collaborating Centre for Research and Training on Viral Zoonoses, King Chulalongkorn Memorial Hospital The Thai Red Cross Society, Bangkok 10330, Thailand; Department of Medicine, Faculty of Medicine, King Mongkut’s Institute of Technology Ladkrabang, Bangkok 10520, Thailand; Department of Preventive and Social Medicine, Faculty of Medicine, Chulalongkorn University, Bangkok 10330, Thailand; Thai Red Cross Emerging Infectious Diseases Health Science Centre, World Health Organization Collaborating Centre for Research and Training on Viral Zoonoses, King Chulalongkorn Memorial Hospital The Thai Red Cross Society, Bangkok 10330, Thailand; Center for Artificial Intelligence in Medicine, Research Affairs, Faculty of Medicine, Chulalongkorn University, Bangkok 10330, Thailand; Center of Excellence in Computational Molecular Biology, Faculty of Medicine, Chulalongkorn University, Bangkok 10330, Thailand; 1 Division of Neurology, Department of Medicine, Faculty of Medicine, Chulalongkorn University, Bangkok 10330, Thailand; Thai Red Cross Emerging Infectious Diseases Health Science Centre, World Health Organization Collaborating Centre for Research and Training on Viral Zoonoses, King Chulalongkorn Memorial Hospital The Thai Red Cross Society, Bangkok 10330, Thailand

**Keywords:** neurofilament, neurofilament light chain, alteration of consciousness, encephalopathy, differential diagnoses

## Abstract

Neurofilament light chain has become a promising biomarker for neuroaxonal injury; however, its diagnostic utility is limited to chronic disorders or specific contexts. Alteration of consciousness is a common clinical problem with diverse aetiologies, many of which require timely diagnoses. We evaluated the value of neurofilament light chain alone, as well as creating diagnostic models, in distinguishing causes of alteration of consciousness. Patients presenting with alteration of consciousness were enrolled. Initial clinical data of each participant were evaluated by a neurologist to give a provisional diagnosis. Each participant subsequently received advanced investigations and follow-up to conclude the final diagnosis. All diagnoses were classified into a structural or non-structural cause of alteration of consciousness. Plasma and cerebrospinal fluid levels of neurofilament light chain were measured. Cerebrospinal fluid neurofilament light chain and other clinical parameters were used to develop logistic regression models. The performance of cerebrospinal fluid neurofilament light chain, the neurologist’s provisional diagnosis, and the model to predict the final diagnosis were compared. For the results, among 71 participants enrolled, 67.6% and 32.4% of their final diagnoses were classified as structural and non-structural, respectively. Cerebrospinal fluid neurofilament light chain demonstrated an area under the curve of 0.75 (95% confidence interval 0.63–0.88) which was not significantly different from a neurologist’s provisional diagnosis 0.85 (95% confidence interval 0.75–0.94) (*P* = 0.14). The multivariable regression model using cerebrospinal fluid neurofilament light chain and other basic clinical data achieved an area under the curve of 0.90 (95% confidence interval 0.83–0.98). In conclusion, neurofilament light chain classified causes of alteration of consciousness with moderate accuracy. Nevertheless, including other basic clinical data to construct a model improved the performance to a level that was comparable to clinical neurologists.

## Teaser

Neurofilament light chain (NFL) is a valuable biomarker for neuroaxonal injury. Ongphichetmetha *et al*. assessed its diagnostic potential and created models to distinguish causes of altered consciousness. Cerebrospinal fluid NFL showed moderate accuracy, and the model using clinical data matched neurologists’ diagnoses. These findings underscore its use in consciousness diagnostics.

## Graphical Abstract

**Graphical Abstract:**
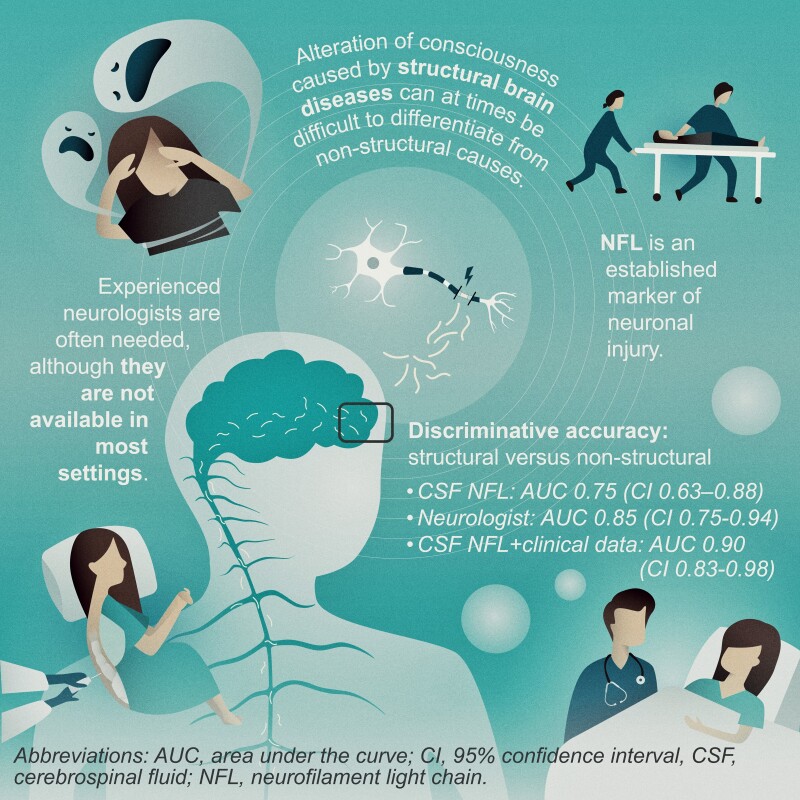

## Introduction

Alteration of consciousness (AOC) is common in both emergency and inpatient settings.^1^ Whereas the mortality rate increases with delayed appropriate treatment, accurate diagnosis of AOC is challenging and time-consuming due to a large range of aetiologies.^1,2^ The most frequent diagnoses causing AOC are primary CNS disorders, intoxication, organ dysfunction and endocrine/metabolic diseases.^1,3^ Consequently, they can be divided into two major categories: structural and non-structural.^4^ It is often necessary to determine whether a patient is suffering from a structural CNS disorder that confers neuronal injury or a non-structural disorder that causes neuronal dysfunction in order to narrow down the differential diagnoses, choose appropriate investigations and prompt initial treatment.

Neurofilament is a class of scaffolding proteins of the neuronal cytoskeleton which provides structural stability to neurons and enables radial growth of axons.^5^ Upon axonal or neuronal damage, neurofilament is released into the extracellular space and subsequently into the CSF and blood. Consequently, neurofilament may represent a biomarker for neuroaxonal injury.^6^ Because of technical reasons, reliable assessment of neurofilament has long been limited to measurements in the CSF and is applicable just for patients who undergo lumbar puncture.^7^ Recently, a single-molecule array (Simoa) has been developed as an ultrasensitive assay that allows quantification of neurofilament in blood with high sensitivity.^8^

Studies generally found that neurofilament level is elevated in structural CNS disorders. Most of them were done on chronic neurological disorders such as multiple sclerosis, neurodegenerative dementias, amyotrophic lateral sclerosis, atypical Parkinsonism and peripheral neuropathies.^5,6,9-15^ For acute CNS disorders, e.g. stroke, encephalitides and traumatic brain injury, studies focused on severity prediction and long-term prognostication,^16-19^ although few diagnostic studies had been done in the most highly selected participants.^19,20^ In traditionally non-structural disorders, neurofilament can be elevated, but in only a few does it reach levels comparable to structural disorders.^21^ For instance, while blood neurofilament is elevated in primary psychiatric disorders (PPD) such as depression and schizophrenia,^22^ it can discriminate frontotemporal lobar degeneration from PPD with high accuracy.^23^ Physiological disturbances insufficient to cause neuronal injury do not seem to affect the neurofilament level. In the youngest patients with presumably the least underlying neuropathology, seizures are not associated with the elevation of this biomarker.^24^ How neurofilament might be implemented to clinical application in aetiology identification has been proposed;^25^ nonetheless, there has been no study directly evaluating the clinical use of neurofilament in patients presenting with AOC.

In the present study, we explore the value of the neurofilament light chain (NFL), which is one of the neurofilament subunits in the CSF and plasma, as a diagnostic biomarker in the aetiology differentiation of AOC. Our hypothesis is that NFL levels will be higher in patients suffering from structural causes of AOC. We expect it to be a diagnostic test with a higher performance than the conventional method, which is the combination of history taking, physical examination, initial laboratory investigation and initial brain imaging. We designed a diagnostic study that aims to use NFL to classify patients presenting with AOC into structural or non-structural causes.

## Materials and methods

### Study design and population

A prospective study at King Chulalongkorn Memorial Hospital was conducted between December 2020 and December 2021. The inclusion criteria were patients aged over 15 years who presented with AOC or developed AOC during admission, and their primary physicians decided to perform a lumbar puncture. Here, AOC is defined as either decreased level or content of consciousness. Decreased level of consciousness can range from drowsy to comatose or Glasgow coma scale <15, whereas impaired content of consciousness refers to any domain including, but not limited to, orientation, attention, speech, perception and thinking. The exclusion criteria were a history of CNS disorders within 1 year or an established neurodegenerative disease. The patients who met the eligibility criteria were consecutively enrolled in the study. History taking, complete neurological examination as well as general physical examination were done in all participants at enrolment (i.e. CSF collection). Basic investigations, such as blood chemistry and neuroimaging, were performed, as well as routine CSF analysis.

A power calculation to determine the appropriate sample size for the study was performed. The calculation was based on the area under the receiver operating characteristics curve (AUC) of 0.96,^26^ for distinguishing boxers from controls, and the prevalence of structural aetiology, which was reported to be 42%.^1^ The power calculation was conducted using the presize package in R. Based on the power calculation, it was determined that a total of 71 participants would need to be enrolled in this study to achieve sufficient statistical power. This study followed the Standards for Reporting of Diagnostic Accuracy (STARD) reporting guideline.^27^ The protocol of this study was not preregistered in a publicly accessible source. However, the complete study protocol is available upon reasonable request from the ethics committee.

### Standard protocol approvals and registration

The institutional review boards of Faculty of Medicine, Chulalongkorn University, Bangkok, Thailand, approved the collection and the use of human subjects for this study (Med Chula IRB no. 930/63). All the methods in this study were performed in accordance with the Declaration of Helsinki. Written informed consent was obtained from all participants or, if incapable, from their proxy, prior to the inclusion in the study.

### Biochemical procedures

Ten millilitres of CSF were collected into polypropylene tubes (Nunc™, cat. no. 339650), whereas blood samples were collected by venipuncture into ethylenediaminetetraacetic acid tubes (BD Vacutainer®, SKU 367863). All samples were centrifuged (10 min at 25°C 2000 × g), separated, divided into aliquots and stored at −80°C within 1 h of the collection according to consensus protocol pending for biochemical analysis.^28^ The CSF NFL levels were measured with enzyme-linked immunosorbent assay (ELISA) (NF-Light™ ELISA; Tecan, cat. no. 30112458) according to the manufacturer’s recommendations. NFL levels in plasma were measured using the Simoa platform (Quanterix, cat. no. 103400). All samples were analysed in duplicates (CSF) or triplicates (plasma) using the same batch of reagents by two certificated laboratory technicians who were blinded to clinical information.

### Diagnostic assessment protocol

#### Conventional method

All clinical, routine laboratory and initial neuroimaging (usually computed tomography) information available prior to lumbar puncture plus routine CSF analysis were assessed by the attending neurology consultant to give a *provisional diagnosis*. We mark this time point for determining the diagnosis to make a comparison with CSF NFL relevant.

#### Reference method

Most participants received further investigations, such as CSF detection of microbial nucleic acids using polymerase chain reaction (PCR), autoantibodies, brain MRI, EEG or tissue diagnosis, which were decided by the neurology consultant (full list provided as Supplementary material). Complicated cases were discussed extensively in a weekly conference by the neurology faculties regarding the possible diagnoses and proper investigations. Each participant’s clinical course and response after receiving treatment for the corresponding diagnosis were monitored. After 12 weeks of follow-up evaluation, the investigators concluded the *final diagnosis* for each participant. All diagnoses were finalized prior to the quantification of the NFL (i.e. blinded).

The provisional and final diagnoses given were then classified into structural or non-structural causes based on the known pathophysiology of diseases.^4^ Septic encephalopathy was traditionally considered non-structural; however, a study reported that it accompanies unequivocal elevation of NFL,^29^ so its categorization into the structural cause was assumed a priori for this study.

### Statistical analysis

Demographic characteristics, medical history, physical examination and diagnostic parameters were summarized as median [interquartile range (IQR)] for continuous variables and number (percentage) for categorical variables, respectively. The final diagnosis, categorized as structural or non-structural, served as the primary outcome. Shapiro–Wilk test was used to assess the normality of data. Acknowledging that the NFL level depends on multiple other factors (such as age or time from onset to measurement), we also tried to predict the final diagnosis category using logistic regression models.

#### Data preprocessing for model development

For practicality, independent variables included were diagnostic parameters that are available in most clinical settings. They included age, body mass index (BMI), number of days after onset, Glasgow coma scale score (GCS), presence of abnormal neurological examination (yes/no), CSF abnormality (normal/abnormal), open pressure, CSF leukocyte count, CSF erythrocyte count, CSF protein, CSF glucose, plasma glucose and CSF NFL. Continuous numerical features were log-transformed, which is generally recommended in intensity and gene and protein expression data. Features with missing values were imputed with the average values. Finally, the values of each feature were standardized to make their mean zero and their standard deviation one. As the data set size is small, 2-fold cross-validation was used to tune the logistic model and to evaluate the performance to ensure that the validation set was large enough (*n* = 35 for training, *n* = 36 for validation). The 2-fold cross-validation process was repeated 10 times to estimate the variability of performance scores.

#### Logistic regression model development

Multivariable logistic regression with LASSO (L1) and ridge (L2) regularization was used to construct the predictive model. The regularization strength varied from 10^−2^ to 10^4^. L1 and L2 regularization were used separately in our analysis. The final model selected for our study utilized L2 regularization. The best model was selected based on the average precision score on the validation sets. Recursive feature elimination was performed to identify the best feature set by removing the feature with the lowest absolute coefficient from consideration and monitoring the average precision score. In addition to average precision, AUC, accuracy and F1, which is the harmonic mean of precision and recall, were also calculated.

#### Comparisons of diagnostic methods

Comparisons of the AUC between CSF NFL alone, the conventional method (neurologist’s provisional diagnosis) and the regression model (NFL plus a few other variables) were performed using DeLong statistics. Statistical significance was defined as a two-sided *P*-value of <0.05. Analysis was performed using Stata 17 (StataCorp, College Station, TX) and Python programming language version 3.8.

## Results

### Participant characteristics

Seventy-one participants were included in our study. The median age was 57 years (range: 15–93 years), and 37 participants were female (52.1%). There was no significant difference in age, BMI and initial GCS between the definite structural group and the definite non-structural group. The number of days from symptoms onset to enrolment of the structural group and the proportion of abnormal neurological examination, CSF profile and initial neuroimaging were significantly different between the diagnostic groups (Table 1).

**Table 1:** Demographic characteristics and clinical variables of the participants

	Overall (*n* = 71)		Structural group (*n* = 48)		Non-structural group (*n* = 23)		
Characteristics	*N*	%	*n*	%	*N*	%	*P*-value
Median (IQR) age, years	57 (28.5–74.5)		57.5 (29.8–74.3)		56 (23.5–73.5)		0.45^a^
Gender							0.126^b^
Female	37	52.1	22	44.8	15	65.2	
Male							
Median (IQR) BMI, kg/m^2^	21.0 (18.7–24.8)		22.2 (18.6–24.7)		21.3 (19.1–24.5)		0.931^a^
Median (IQR) days after symptoms onset to enrolment	7 (3.5–30)		13.5 (5.8–67.5)		3 (1–7)		0.00^a^
Median (IQR) initial GCS	14 (13–15)		14 (13–14.3)		14 (14–15)		0.163^a^
Abnormal neurological examination	26	36.6	22	45.8	4	17.4	0.035^b^
Abnormal basic blood laboratory investigation^d^	9	12.7	7	14.6	2	8.7	0.71°^c^
Abnormal CSF profile^e^	20	28.2	19	39.6	1	4.3	0.002^b^
CSF leukocyte count >10 cells/mm^3^	16	22.5	15	31.3	1	4.3	0.037^b^
Median (IQR) CSF protein, mg/dL	44.5 (29.2–74.)		55.0 (40.9–85.25)		31.95 (24.70–39.17)		0.00^a^
Abnormal initial neuroimaging^f^	16	22.5	15	31.3	1	4.3	0.011^b^
Mortality	10	14.1	10	20.8	0	0	0.025^c^
Provisionally misdiagnosed	9	12.6	4	8.3	5	21.7	0.138^c^
Median (IQR) CSF NfL	2759.5 (606.6–6891.4)		3172.1 (1360.6–8705.8)		624.6 (177.2–2618.7)		0.001^a^

BMI, body mass index; CSF, cerebrospinal fluid; GCS, Glasgow coma scale; NfL, neurofilament.

^a^Mann–Whitney U-test.

^b^Chi-square test.

^c^Fisher’s exact test.

^d^Basic blood laboratory investigation: complete blood count (CBC), blood urea nitrogen (BUN), creatinine, electrolytes (sodium, potassium, chloride, bicarbonate, calcium, phosphate, and magnesium), and liver function test [aspartate aminotransferase (AST), alanine aminotransferase (ALT), total bilirubin, direct bilirubin, total protein, globulin, and albumin].

^e^Abnormal CSF profile was defined as CSF leukocyte count >10 cells/mm^3^ and/or CSF protein > 50 mg/dL.

^f^Abnormal initial neuroimaging was defined as brain CT reports that were reviewed by neuroradiologists and found to exhibit abnormalities known to be associated with AOC.

Forty-eight participants (67.6%) received the diagnoses that are classified into the structural group. The diagnoses were septic encephalopathy (*n* = 8), acute ischaemic stroke (*n* = 5), autoimmune encephalitis (*n* = 4), viral encephalitis (*n* = 4), unspecified neurodegenerative dementia (*n* = 4), cryptococcal meningitis (*n* = 3), brain metastasis (*n* = 3), post-infectious encephalitis (*n* = 2), CNS lymphoma (*n* = 2), Creutzfeldt–Jakob disease (*n* = 2), cerebral amyloid angiopathy (*n* = 2), bacterial meningitis (*n* = 1), tuberculous meningitis (*n* = 1), mitochondrial encephalomyopathy, lactic acidosis and stroke-like episodes (*n* = 1), pachymeningitis secondary to systemic autoimmune disease (*n* = 1), CNS vasculitis (*n* = 1), aseptic meningitis (*n* = 1), non-tuberculous mycobacterial encephalitis (*n* = 1), posterior reversible encephalopathy syndrome with intracerebral haemorrhage (*n* = 1) and germinoma (*n* = 1).

Twenty-three participants were ultimately classified into the non-structural group. The diagnoses were seizures (*n* = 8), psychiatric disorders (*n* = 7), metabolic encephalopathy (*n* = 5), drug intoxication (*n* = 2) and Wernicke encephalopathy (*n* = 1). Details on the results of the investigations of each participant were included in Supplementary Table 1.

### NFL levels and its performance as a diagnostic test

**Figure 1:**
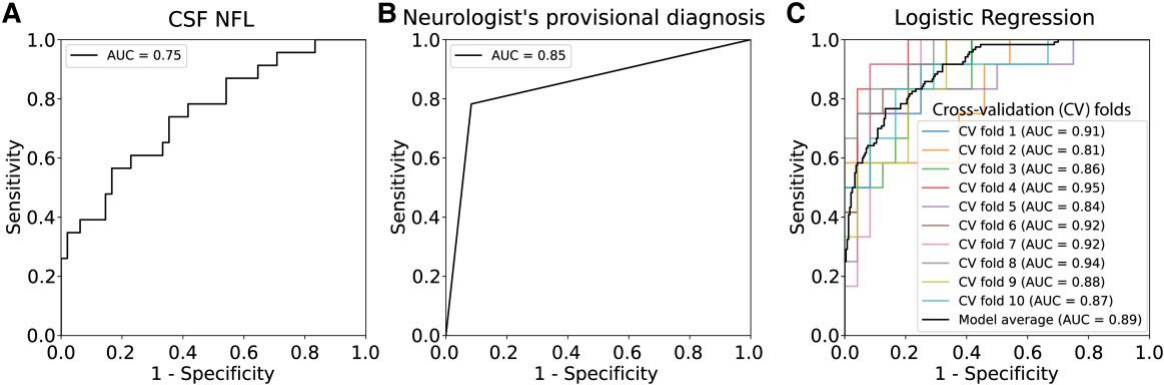
**Receiver operating characteristics (ROC) curves for each aetiologic classification model.** (**A**) ROC curve for the CSF NFL (*n* = 71). An area under the curve is 0.75 (95% confidence interval 0.63–0.88). (**B**) ROC curve for neurologist’s provisional diagnosis (*n* = 71). An area under the curve is 0.85 (95% confidence interval 0.75–0.94). (**C**) ROC curve for the best logistic regression model. ROC curves from 10 repeats of 2-fold cross-validation (*n* = 35 for training, *n* = 36 for validation) were shown seperately, with the thick dark line representing the average over 10 repeats. Abbreviations: AUC, area under the curve; CV, cross-validated.

CSF NFL concentrations were significantly higher in the structural group (median 3172.1 versus 624.6 pg/mL, *P* = 0.001) (Table 1). CSF NFL levels alone demonstrated a moderate AUC of 0.75 (95% CI 0.63–0.88) for distinguishing the structural group from the non-structural group (Fig. 1A). The optimal cut-off value, determined to be 808.8 pg/mL, achieved a Youden index of 0.4, indicating a sensitivity of 83.3%, specificity of 56.5%, positive predictive value of 80% and negative predictive value of 61.9%. The Supplementary material provides additional comprehensive diagnostic parameters for reference, as well as a sensitivity analysis using various methods to identify the optimal cut-off (Supplementary Tables 2 and 3; Supplementary Figs. 1 and 2). In contrast, the conventional method, the neurologist’s provisional diagnosis, resulted in a sensitivity of 91.7% and specificity of 78.2% which can be transformed into the AUC of 0.85 (95% CI 0.75–0.94) (Fig. 1B). There was no significant difference between the diagnostic performance of CSF NFL and the conventional method (AUC 0.75 versus 0.85, *P* = 0.14). Plasma NFL was available in 62 participants, where it demonstrated only a moderate correlation with CSF NFL (Rho = 0.57, *P* < 0.001) and a relatively low AUC of 0.60 (95% CI 0.43–0.76). Therefore, we did not use plasma NFL in any further analysis.

Among 37 participants (52.1%) who were younger than 60 years old, 25 and 12 participants were classified into the structural group and non-structural group, respectively; CSF NFL showed a larger AUC (0.89, 95% CI 0.79–0.99). In comparison, the conventional method resulted in a sensitivity of 92% and specificity of 100% which can be transformed into an AUC of 0.915.

### Model for aetiologic classification

**Figure 2:**
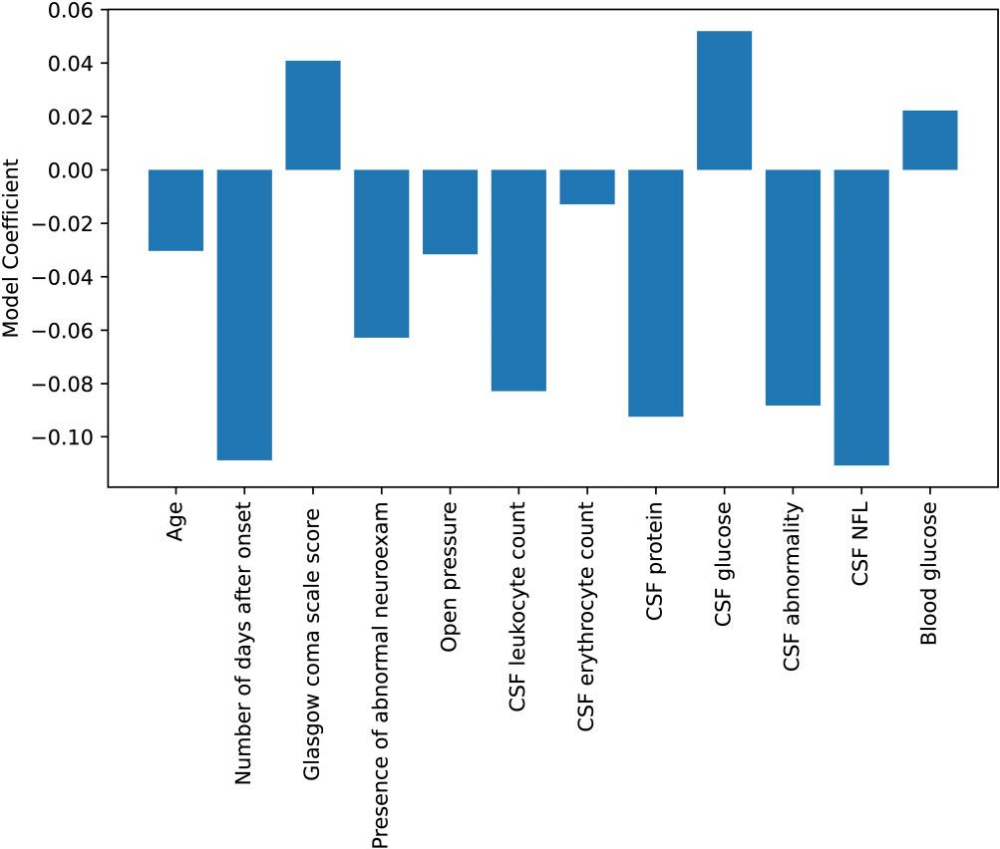
**Average coefficient values for the best logistic regression model for aetiologic classification.** The model was trained on standardized data where the standard deviation of each input feature was scaled to 1. Coefficient values were averaged over 10 repeats of 2-fold cross-validation. CSF NFL was assigned the largest average coefficient magnitude.

An initial multivariate logistic regression model for aetiologic classification was developed without using the neurologist’s provisional diagnosis to evaluate the predictive power of CSF NFL and other diagnostic parameters which are easy to acquire. There were four missing BMI values and three missing plasma glucose values. After performing feature selection with recursive feature elimination, only BMI was removed from the input feature set. The best multivariate logistic regression model achieved an AUC of 0.90 (95% CI 0.83–0.98) (Fig. 1C). The equation used in this model was included in the Supplementary material. The inclusion of a neurologist’s provisional diagnosis into this model increased the average AUC by 0.05 although this was not statistically significant (*P* = 0.39). CSF NFL has the highest impact on the model (i.e. assigned the highest absolute coefficient value), followed by the number of days after onset, CSF protein and CSF abnormality, respectively (Fig. 2).

## Discussion

NFL has emerged as a robust biomarker for neuroaxonal injury. However, its level only alters days after the onset of such an injury. Therefore, its diagnostic use is better established in diseases with chronic ongoing neuroaxonal loss, in which the rate of its release reaches a steady state. Moreover, its level in healthy people varies greatly and increases with age, vascular comorbidities and undiagnosed neurodegenerative pathology. It is difficult to determine a baseline level in an individual patient, particularly in an elderly individual. Using NFL as a diagnostic biomarker in non-chronic illnesses is therefore complicated.

In this study of prospectively enrolled patients with AOC, we aimed to use NFL as a diagnostic biomarker for differential diagnosis. As is well-known, AOC often arises from widespread neuronal dysfunction. In cases where the underlying cause of this dysfunction is a structural brain disease, a high degree of neuroaxonal injury is typically anticipated. The rationale for classifying the diagnoses into two groups (structural versus non-structural) is that it confers immediate application and affects further management. AOC resulting from structural causes typically necessitates immediate investigation to establish a definitive diagnosis, determine appropriate empirical treatment and potentially consider surgical interventions. Conversely, the diagnostic process for AOC stemming from non-structural causes is often more protracted and comprehensive.^4^ To illustrate this point, consider a young female presenting with acute psychosis. If the underlying cause is determined to be autoimmune encephalitis, treatment would involve intravenous corticosteroids. However, if the primary diagnosis is a PPD, treatment may primarily consist of antipsychotic drugs, as the use of corticosteroids can exacerbate the symptoms.^30^

While CSF NFL alone predicts structural diagnoses relatively well for participants under 60 years of age, its performance with all participants is only moderate. This finding is not unexpected, considering the well-known variability of CSF NFL levels in older adults, especially in the presence of cerebrovascular burden. However, we tried to overcome these difficulties with a data-driven approach using additional clinical information readily available in most settings. Our model was able to distinguish structural CNS disorders with a high accuracy similar to, if not better than, medical school neurology consultants. Apart from NFL, the model heavily depends on routine CSF indices. This is probably because diseases primarily involving the meninges may affect consciousness without causing much neuroaxonal injury, and therefore, the model relies on CSF abnormality as a biomarker for such diagnoses. The fact that CSF NFL had the highest impact on the model despite being the only parameter that was truly blinded (i.e. no risk of incorporation bias) demonstrated that the success of this model was not due to the statistical handling of routine clinical data alone. Once NFL becomes widely available, like cardiac troponins, our model will provide a useful tool not only for resource-limited settings where a neurologist is not available but also possibly for tertiary care settings where neurologists can benefit from this complex biomarker. Indeed, point-of-care testing for a related biomarker, the astrocytic intermediate filament glial fibrillary acidic protein, has been developed and can deliver results within 15 min.^31^ To demonstrate how this model can be implemented in aiding the differential diagnosis of AOC, we created an online tool at https://trceid.org/diagNFL, in which NFL-based individual probability of structural aetiology of AOC can be calculated.

There are some limitations to our study. Firstly, we used a relatively small sample size for a prediction model and still lacked external validation. This is mainly because our primary outcome was to test CSF NFL alone as a diagnostic marker, and our sample size was calculated for such. Consequently, until successful verification in an independent cohort, the implications of our initial findings probably do not immediately translate to clinical practice. Instead, the impressive accuracy of our model suggests that it holds considerable potential for exploring novel approaches to utilizing this biomarker. Moreover, as the molecular underpinning of NFL release is more extensively elucidated, it opens the possibility of incorporating other explanatory variables into our model, potentially leading to further improvements in diagnostic performance. The second limitation is that the reference diagnosis in this study is the practical final diagnosis which, for some participants, is not the criteria-based definite diagnosis. While we acknowledge that this affects the credibility of our study, we realized that most real-world neurological diagnoses are not criteria-based. Limiting our study analysis to those with definite diagnoses would substantially deteriorate the applicability of our study. On the other hand, we are certain that we had provided sufficient diagnostic tests and follow-up to declare our final diagnosis. In our consecutively enrolled participants, the duration of AOC prior to lumbar puncture differed significantly between the two diagnostic groups. While we believe that this represented a real-life clinical practice, this discrepancy can influence NFL levels. Because NFL is a dynamic biomarker which levels tend not to peak until the second week, our findings may have been affected. Consequently, caution should be exercised when interpreting NFL levels in acute settings, taking into account the potential impact of the time elapsed between symptom onset and CSF collection. Finally, our study started prior to the publication of the guidance on the general implementation of NFL to symptoms of unknown aetiology, which recommended serial measurement for patients without a baseline level.^25^ It is interesting to see how following the guidance would improve the performance of the NFL in this study.

In conclusion, CSF NFL can distinguish structural aetiology from non-structural causes of AOC with moderate accuracy. Nevertheless, adding other clinical data to construct a data-driven model improved the performance to a level that matches clinical neurologists. CSF NFL is a useful biomarker for differential diagnosis of AOC that can be implemented in primary care and resource-limited settings.

## Supplementary material

Supplementary material is available at *Brain Communications* online.

## Supplementary Material

fcad278_Supplementary_Data

## Data Availability

The original contributions presented in the study are included in this article/Supplementary material; further inquiries can be directed to the corresponding author. The data are not publicly available due to privacy or ethical restrictions.
