# Supplementary material for: Neurofilament light chain for classifying the aetiology of alteration of consciousness
Source: Brain Commun. 2023 Oct 18;5(6):fcad278. doi: 10.1093/braincomms/fcad278 (PMC10629465; doi:10.1093/braincomms/fcad278)
Supplement: fcad278_Supplementary_Data [file fcad278_supplementary_data.pdf]

Supplementary materials

Supplementary Table 1 Details on the results of further investigations conducted on each participant to conclude the *final diagnosis*

No	Age	Structural	Final diagnosis	Investigation
1	20s	Yes	Viral encephalitis	- CSF profile: Lymphocytic pleocytosis<br>- CSF Bacterial culture: No growth<br>- CSF ME panel: Not detected<br>- CSF PCR for HSV 1-2 and VZV: Not detected<br>- CSF PCR for MTBC: Negative<br>- CSF Autoimmune encephalitis antibodies panel: Negative<br>- MRI Brain with Gad: Normal
2	80s	Yes	Septic encephalopathy	- Urine Bacterial culture: Enterococcus faecium<br>- CSF profile: Normal<br>- CSF Bacterial culture: No growth<br>- CSF ME panel: Not detected<br>- CT brain without contrast: Aging brain<br>- EEG: Moderate diffuse encephalopathy
3	80s	No	Baclofen side effect	- CSF profile: Normal<br>- CSF Bacterial culture: No growth<br>- MRI Brain without Gad: Normal
4	70s	Yes	MELAS	- MRI brain with Gad: Cerebral swelling with gyral expansion with gyral enhancement involving entire right cerebral hemisphere with some faint subtle restricted diffusion and T2/FLAIR hyperSI involving subcortical white matter of the right occipital lobe<br>- CSF Lactate: 2 mmol/L<br>- Blood for Whole mitochondrial genome: No disease associated mutation<br>- EEG: Intermittent rhythmic delta activity at left frontotemporal region<br>- CSF profile: Normal<br>- CSF ME panel: Not detected<br>- CSF Autoimmune encephalitis antibodies panel: Negative
5	70s	No	Epilepsy	- CSF profile: Normal<br>- MRI Brain without Gad: Old lacune at left caudate and cerebellar hemisphere with brain atrophy<br>- EEG: Occasional TIRDA at left temporal region
6	70s	Yes	CJD	- MRI Brain with Gad: Cortical ribbon sign with restricted diffusion at left parieto-temporo-occipital region<br>- EEG: Periodic sharp wave complex at left frontotemporal region<br>- CSF T-tau/P-tau: >1600/43.7 (very high)
7	70s	No	Delirium	- CSF profile: Normal<br>- MRI Brain: Normal
8	60s	Yes	Acute left MCA infarction	- MRI brain: Acute to subacute left MCA infarction

9	80s	Yes	Cerebral amyloid angiopathy related inflammation	- MRI brain with Gad: Convexal subarachnoid hemorrhage along sulci of left parieto-occipital lobe with severe microbleeds along sulcal surface of bilateral cerebral hemisphere, cortical gray and white matter of left parieto-occipital lobe, and right thalamus, probably inflammatory cerebral amyloid angiopathy (CAA)<br>- EEG: Cotinuous 1.5-2 Hz periodic discharge prominent at left cerebral hemisphere without evolution and occasional sharp and slow at right cerebral hemisphere
10	40s	Yes	Tuberculous meningoencephalitis	- CSF Mycobacterium culture: Mycobacterium tuberculosis complex<br>- MRI Brain with Gad: Leptomeningeal enhancement surrounding brainstem, cortical sulci of right medial temporal lobe, cisternal segment of bilateral trigeminal nerves, suprasellar cistern and pituitary stalk with T1 hyperintensity along perivascular spaces of bilateral lentiform nuclei and suspected T2/FLAIR hyperintensity at right postero-medial temporal lobe, compatible with tuberculous meningoencephalitis
11	20s	No	Bipolar disorder	- CSF profile: Normal<br>- CSF Bacterial culture: No growth<br>- CSF ME panel: Not detected<br>- CSF PCR for HSV 1-2: Not detected<br>- CSF and Blood Autoimmune encephalitis antibodies panel: Negative<br>- MRI Brain with Gad: Normal<br>- EEG: Mild focal cerebral dysfunction at right paracentral area
12	60s	No	Epilepsy	- CSF profile: Normal<br>- CSF Bacterial culture: No growth<br>- MRI Brain with Gad: Normal<br>- EEG: 1 episode of rhythmic delta activity at bilateral paracentral area evolute to generalized rhythmic delta activity, with clinical focal motor seizure with secondary generalized clonic seizure
13	20s	No	Bipolar disorder	- CSF profile: Normal<br>- CSF Bacterial culture: No growth<br>- CSF ME panel: Not detected<br>- CSF and Blood Autoimmune encephalitis antibodies panel: Negative<br>- MRI Brain with Gad: Normal
14	10s	Yes	Suspected post infectious meningoencephalitis on top toxic metabolic leukoencephalopathy	- CSF profile: Lymphocytic pleocytosis<br>- CSF Bacterial culture: No growth<br>- CSF ME panel: Not detected<br>- CSF PCR for HSV 1-2 and VZV: Not detected<br>- CSF PCR for MTBC: Negative<br>- CSF Autoimmune encephalitis antibodies panel: Negative<br>- MRI Brain with Gad: Small confluent abnormal restricted diffusion without abnormal intensity in other imaging sequence in deep white matter of bilateral frontoparietal region, and a few small foci in right sided genu of corpus callosum and right parieto-occipital deep white matter. Differential diagnosis include acute leukoencephalopathy with restricted diffusion (ALERD), viral encephalitis, and toxic or inherited metabolic

				encephalopathy. Faint cerebral sulcal leptomeningeal enhancement on post contrast FLAIR images, leptomeningeal process is suspected.
15	50s	Yes	Pachymeningitis secondary to autoimmune disease (Granulomatosis polyangiitis)	- CSF profile: Normal<br>- CSF Bacterial culture: No growth<br>- CSF ME panel: Not detected<br>- CSF PCR for MTBC: Negative<br>- CSF VDRL: Non-reactive<br>- MRI Brain with Gad: An irregular-thickened heterogeneous-enhancing dura along the falx cerebri, more pronounce at posterior aspect, probably hypertrophic pachymeningitis
16	50s	Yes	Leptomeningeal metastasis	- MRI Brain with Gad: Diffuse leptomeningeal enhancement in both supra and infratentorial regions, as well as enhancement along multiple cranial nerves, most favorable leptomeningeal carcinomatosis<br>- CSF Cytospin: Non-hematopoietic cells were seen, suspicious of CNS metastasis
17	50s	Yes	Acute ischemic stroke	- MRI Brain with Gad: Only a spot of restricted diffusion of brain parenchyma in each centrum semiovale seen on DWI and ADC map, suspected areas of acute to subacute infarcts<br>- CSF profile: Normal<br>- CSF Bacterial culture: No growth<br>- CSF PCR for MTBC: Negative
18	40s	Yes	Early onset Alzheimer's disease	- MRI Brain: Cerebral volume loss with mildly increased degree in bilateral hippocampus<br>- EEG: Focal cerebral dysfunction at bilateral temporal area, more prominent over right side<br>- CSF profile: Normal<br>- CSF Bacterial culture: No growth<br>- CSF ME panel: Not detected<br>- CSF PCR for MTBC: Negative<br>- CSF and Blood Autoimmune encephalitis antibodies panel: Negative<br>- CSF and Blood Paraneoplastic antibodies panel: Negative
19	60s	Yes	Viral meningoencephalitis	- CSF profile: Lymphocytic pleocytosis<br>- CSF Bacterial culture: No growth<br>- CSF ME panel: Not detected<br>- CSF PCR for HSV 1-2: Not detected<br>- CSF PCR for MTBC: Negative<br>- CSF Cryptococcal antigen: Negative<br>- MRI Brain with Gad: Normal<br>- EEG: NCSE
20	40s	Yes	Brain metastasis	- MRI Brain with Gad: Small enhancing nodules at bilateral cerebral and cerebellar hemisphere, bilateral basal ganglia, bilateral thalami, splenium of corpus callosum, left cerebral peduncle, representing brain metastasis

21	20s	Yes	Cryptococcal meningitis with hydrocephalus	- CSF ME panel: Cryptococcal neoformans detected<br>- CSF Cryptococcal antigen: Positive titer 1:2048<br>- CSF Bacterial culture: Cryptococcal neoformans slight growth<br>- CSF PCR for MTBC: Negative<br>- Hemoculture: Cryptococcal neoformans<br>- CT Brain with contrast: No demonstrable intracranial abnormality<br>- EEG: Focal cerebral dysfunction at left cerebral hemisphere
22	70s	Yes	DLBCL with CNS involvement	- CSF profile: Mild pleocytosis with elevated protein<br>- MRI Brain with Gad: Multifocal ill-defined patchy T2/FLAIR hyperintense lesions with nodular and irregular linear enhancement involving bilateral frontal, parietal and temporal deep and periventricular white matter, deep grey structures, midbrain, dorsal pons, bilateral superior cerebellar peduncles, medial aspect of both middle cerebellar peduncles and bilateral peri-fourth ventricular cerebellar region with enhancement along medullary veins and perivascular spaces distribution and a few foci of restricted diffusion, probably CNS involvement of lymphoma<br>- CSF Cytospin: No cell
23	60s	Yes	CNS lymphoma	- CSF profile: Mild pleocytosis with elevated protein<br>- MRI Brain with Gad: Newly seen several foci of T2/FLAIR hyperintense lesions with contrast enhancement and restricted diffusion in medial aspect of bilateral cerebellar hemispheres and cortex of bilateral occipital, right parietal and left temporal lobe, probably cerebral lymphoma<br>- CSF Cytospin: No cell<br>- CSF Cytology: No cell
24	20s	Yes	Tuberculous vasculitis with multiple acute lacunar infarction	- MRI Brain with Gad: Multiple areas of leptomeningeal enhancement, multiple nodular enhancement along right lateral ventricle and 4th ventricle, small enhancing nodules at anterior left external capsule and right anterior temporal lobe, infection/inflammation (Ddx including TBb and/or cryptococcosis) and moderate/severe stenosis of intracranial arteries including occlusion of distap P1 segment of left PCA and multiple small acute infraction, suspicious of vasculitis and/or vasospasm<br>- CSF PCR for MTBC: MTBC DNA detected<br>- CSF Mycobacterium culture: Mycobacterium tuberculosis
25	50s	Yes	AntiLGI1 encephalitis	- CSF autoimmune encephalitis antibodies panel: Anti-LGI1 antibodies positive<br>- MRI Brain with Gad: HyperSI T2/FLAIR at right mesial temporal region<br>- EEG: TIRDA at right temporal region
26	30s	No	Metabolic encephalopathy	- CSF profile: Normal<br>- CSF Bacterial culture: No growth<br>- CT Brain with contrast: Normal
27	70s	Yes	CJD	- MRI Brain with Gad: Restricted diffusion with hyperSI T2/FLAIR of bilateral parietooccipital lobe and left frontal cortex<br>- EEG: Periodic sharp wave complex at left temporo-occipital region<br>- CSF T-tau/P-tau: Very high

28	50s	Yes	Anti-GAD65 with borderline Anti-Ma2 with atypical stiff person syndrome	- CSF autoimmune encephalitis antibodies panel: Anti-GAD65 and anti-ma2 positive<br>- MRI Brain with Gad: Midbrain atrophy with mild thinning of the superior colliculus
29	70s	No	Status epilepticus provoked by hyperglycemia	- CSF profile: Normal<br>- CSF Bacterial culture: No growth<br>- CSF PCR for MTBC: Negative<br>- CT Brain with contrast: Normal
30	70s	No	Hepatic encephalopathy	- CSF profile: Normal<br>- CSF Bacterial culture: No growth<br>- CSF ME panel: Not detected<br>- CSF PCR for MTBC: Negative<br>- CT Brain with contrast: Normal
31	70s	Yes	Bacterial meningitis with acute ischemic stroke	- CSF profile: Pleocytosis with PMN predominate with elevated protein and low glucose<br>- CSF Bacterial culture: Bacillus species moderate growth<br>- CSF ME panel: Not detected<br>- CSF PCR for MTBC: Negative<br>- MRI Brain with Gad: Several small non-enhancing T2/FLAIR hyperintense foci, some with restricted diffusion and some with enhancement, scattering in cortical-subcortical region and deep and white matter of left parieto-occipital lobes, genu of left internal capsule and left thalamus, probably acute ot subacute infarction
32	80s	Yes	Septic encephalopathy	- CSF profile: Normal<br>- CSF Bacterial culture: No growth<br>- CT Brain with contrast: Normal
33	30s	Yes	Septic encephalopathy	- CSF profile: Normal<br>- CSF Bacterial culture: No growth<br>- CSF ME panel: Not detected<br>- CT Brain with contrast: Normal
34	80s	Yes	Septic encephalopathy	- CSF profile: Normal<br>- CSF Bacterial culture: No growth<br>- CT Brain with contrast: Normal
35	20s	No	Metabolic encephalopathy	- CSF profile: Normal<br>- CSF Bacterial culture: No growth<br>- CT Brain with contrast: Normal
36	70s	Yes	Psychiatric disease in elderly	- CSF profile: Normal<br>- CSF Bacterial culture: No growth<br>- CSF ME panel: Not detected<br>- CSF VDRL: Non-reactive<br>- CSF and Blood Autoimmune encephalitis antibodies panel: Negative<br>- CSF and Blood Paraneoplastic antibodies panel: Negative<br>- CSF and Blood Unclassified antibodies: Negative

				- MRI Brain: Mild diffuse brain atrophy with chronic ischemic white matter change<br>- EEG: Normal
37	90s	Yes	Left ICA stenosis with acute ischemic stroke	- CT brain non contrast and CTA of the brain and neck: Multifocal ill-defined hypodense lesions in subcortical and deep white matter of left inferior frontal lobe and genu of left corpus callosum, probably acute infarction with absent contrast opacification along extra-cranial left ICA at C2 level to intracranial portion at paraclinoid segment with contrast reconstitution at left supraclinoid ICA<br>- CSF profile: Normal<br>- CSF Bacterial culture: No growth
38	80s	Yes	Cerebral amyloid angiopathy	- MRI Brain with Gad: Multiple tiny nodular enhancing foci in bilateral cerebellar hemispheres and right posterior temporal cortex, probably subacute microinfarction with multiple lobar microbleeds in bilateral cerebral hemispheres, and along superficial areas of cerebellar hemispheres and vermis, favored cerebral amyloid angiopathy (CAA)<br>- CSF profile: Normal
39	50s	No	Psychotic disorder	- CSF profile: Normal<br>- CSF Bacterial culture: No growth<br>- CSF ME panel: Not detected<br>- CSF VDRL: Non-reactive<br>- CSF and Blood Autoimmune encephalitis antibodies panel: Negative<br>- CSF and Blood Paraneoplastic antibodies panel: Negative<br>- CSF and Blood Unclassified antibodies: Negative<br>- MRI Brain: Ischemic white matter change
40	80s	Yes	Septic encephalopathy	- CSF profile: Normal<br>- CSF Bacterial culture: No growth<br>- CSF ME panel: Not detected<br>- CT Brain with contrast: Normal
41	80s	Yes	Herpes encephalitis	- CSF profile: Normal<br>- CSF Bacterial culture: No growth<br>- CSF ME panel: HSV-1 detected<br>- CT Brain with contrast: Normal
42	60s	No	Cefazoline intoxication	- CSF profile: Normal<br>- CSF Bacterial culture: No growth<br>- MRI Brain: Mild communicating hydrocephalus
43	20s	No	Acute psychosis	- CSF profile: Normal<br>- CSF Bacterial culture: No growth<br>- CSF ME panel: Not detected<br>- CSF and Blood Autoimmune encephalitis antibodies panel: Negative<br>- CSF and Blood Unclassified antibodies: Negative<br>- CT Brain with contrast: Normal
44	20s	Yes	Cryptococcal meningitis	- CSF Cryptococcal antigen: Positive titer 1:2048<br>- CSF Bacterial culture: Cryptococcal neoformans moderate growth

				- CSF PCR for MTBC: Negative<br>- MRI Brain with Gad: Finding of increased intracranial pressure with thinning pituitary gland, prominent subarachnoid spaces around the optic nerves, vertical tortuosity of the optic nerves, flattening of the posterior sclera, and intraocular protrusion of the optic nerve heads and faint enhancement along bilateral cerebral sulci and cerebellar fissures, could be engorged cortical vessels or leptomeningitis
45	60s	Yes	Post immunization (ChAdOx1-S/nCoV-19) encephalopathy	- CSF profile: Normal<br>- CSF Bacterial culture: No growth<br>- CSF ME panel: Not detected<br>- MRI Brain: Old hemorrhage involving right putamen, external capsule and temporal lobe<br>- EEG: Mild diffuse encephalopathy
46	10s	Yes	Germinoma	- CSF profile: Lymphocytic pleocytosis with elevated protein<br>- CSF Bacterial culture: No growth<br>- CSF Cytology: No atypical cell seen<br>- MRI Brain with Gad: Germ cell tumor at sellar-suprasellar region with CSF seeding
47	70s	No	Epilepsy	- CSF profile: Normal<br>- CSF Bacterial culture: No growth<br>- CSF and Blood Autoimmune encephalitis antibodies panel: Negative<br>- CSF and Blood Unclassified antibodies: Negative<br>- CT Brain with contrast: Normal<br>- EEG: Fast activity at bilateral temporal area, more pronounced on left cerebral hemisphere during event
48	10s	Yes	Disseminated cryptococcosis	- CSF ME panel: Cryptococcal neoformans detected<br>- CSF Cryptococcal antigen: Positive titer 1:2048<br>- CSF Bacterial culture: Cryptococcal neoformans slight growth<br>- CSF PCR for MTBC: Negative<br>- Hemoculture: Cryptococcal neoformans<br>- CT Brain with contrast: A few small ill-defined hypodense lesions involving bilateral caudate head, anterior body of corpus callosum and deep white matter of right frontal lobe without obvious leptomeningeal enhancement
49	70s	Yes	Septic encephalopathy	- CSF profile: Normal<br>- CSF Bacterial culture: No growth<br>- CSF ME panel: Not detected<br>- CT Brain with contrast: Normal
50	20s	Yes	Septic encephalopathy	- CSF profile: Normal<br>- CSF Bacterial culture: No growth<br>- CSF ME panel: Not detected<br>- CSF and Blood Autoimmune encephalitis antibodies panel: Negative<br>- CSF and Blood Unclassified antibodies: Negative<br>- MRI Brain with Gad: Normal

51	20s	Yes	HIV encephalopathy with suspected cerebral toxoplasmosis	- CSF profile: Elevated protein<br>- CSF Bacterial culture: No growth<br>- CSF ME panel: Not detected<br>- CSF PCR for MTBC: Not detected<br>- CSF Cryptococcal antigen: Negative<br>- Serum toxoplasma IgM negative, IgG positive<br>- MRI Brain with Gad: A few contiguous irregular thin-rim enhancing lesions in right frontal white matter, right external and internal capsules and right lentiform nucleus with small internal hemorrhage, as well as rim-enhancing lesions with peripheral restricted diffusion in cortical/subcortical regions of bilateral cerebral hemispheres, left lentiform nucleus, bilateral thalami, bilateral cerebral peduncles, bilateral cerebellar hemispheres and cerebellar vermis, probably hematogenous infectious process such as toxoplasmosis or tuberculosis
52	10s	No	Post SARS-CoV-2 infection encephalopathy	- CSF profile: Normal<br>- CSF Bacterial culture: No growth<br>- CSF ME panel: Not detected<br>- CSF and Blood Autoimmune encephalitis antibodies panel: Negative<br>- CSF and Blood Unclassified antibodies: Negative<br>- MRI Brain with Gad: Small T2/FLAIR hyperintense foci in white matter of bilateral frontal lobes<br>- EEG: Lateralized rhythmic delta activity at right occipital region
53	30s	Yes	PRES	- CSF profile: Normal<br>- CSF Bacterial culture: No growth<br>- CSF PCR for MTBC: Not detected<br>- CT Brain with contrast: Multifocal hypodense lesions with some enhancement involving cortex and subcortical white matter of bilateral frontal, left parietal and bilateral temporooccipital lobes. Differential diagnosis included posterior reversible encephalopathy syndrome (PRES) or post ictal change
54	20s	No	Acute psychosis	- CSF profile: Normal<br>- CSF Bacterial culture: No growth<br>- CSF ME panel: Not detected<br>- CSF PCR for MTBC: Not detected<br>- CSF VDRL: Non-reactive<br>- CSF and Blood Autoimmune encephalitis antibodies panel: Negative<br>- CSF and Blood Unclassified antibodies: Negative<br>- MRI Brain with Gad: Normal
55	50s	Yes	Acute ischemic stroke	- CSF profile: Normal<br>- CSF Bacterial culture: No growth<br>- CSF ME panel: Not detected<br>- CSF PCR for MTBC: Not detected<br>- CSF VDRL: Non-reactive

				- MRI and MRA Brain: Left PCA infarction with moderate to severe stenosis left PCA
56	20s	Yes	Aseptic meningitis	- CSF profile: Elevated protein<br>- CSF Bacterial culture: No growth<br>- CSF ME panel: Not detected<br>- CSF PCR for MTBC: Not detected<br>- CT Brain non contrast: Normal
57	60s	No	Wernicke encephalopathy	- CSF profile: Normal<br>- CSF Bacterial culture: No growth<br>- CSF ME panel: Not detected<br>- CT Brain non contrast: Normal
58	20s	Yes	VZV meningitis	- CSF profile: Normal<br>- CSF Bacterial culture: No growth<br>- CSF ME panel: VZV detected<br>- CSF PCR for VZV: Viral load 31750 copies/ml<br>- CT Brain with contrast: Normal
59	50s	No	Unprovoked seizure	- CSF profile: Normal<br>- CSF Bacterial culture: No growth<br>- CT Brain with contrast: Normal
60	20s	Yes	Anti-NMDAR encephalitis	- CSF profile: Lymphocytic pleocytosis<br>- CSF ME panel: Not detected<br>- CSF and Blood Autoimmune encephalitis antibodies panel: Anti-NMDAR antibodies positive<br>- MRI Brain with Gad: Normal<br>- EEG: Normal
61	10s	No	Unprovoked seizure	- CSF profile: Normal<br>- CSF ME panel: Not detected<br>- CSF and Blood Autoimmune encephalitis antibodies panel: Negative<br>- MRI Brain with Gad: Normal<br>- EEG: Focal IED was seen
62	60s	Yes	Cancer associated ischemic stroke	- CSF profile: Normal<br>- CSF Bacterial culture: No growth<br>- MRI Brain with Gad: Multiple ischemic stroke at bilateral cerebral hemisphere with some gyral hemorrahge and/or cortical laminar necrosis, and gyral enhancement
63	60s	Yes	Lung cancer with brain metastasis	- CT Brain with contrast: Multiple faint hyperdense lesions with faint enhancement and some showing nodularity, symmetrically scattering along bilateral cerebral and cerebellar surfaces and periventricular and deep white matter of bilateral cerebral hemisphere, possibly leptomeningeal metastasis<br>- CSF Cytology: No atypical cell seen

64	80s	Yes	Unspecified neurodegenerative disease	- CSF profile: Normal<br>- CSF Bacterial culture: No growth<br>- CSF VDRL: Non-reactive<br>- CSF and Blood Autoimmune encephalitis antibodies panel: Negative<br>- CSF Total tau: Negative<br>- CSF P-tau: Negative<br>- MRI Brain with Gad: Small vessel disease with bilateral hippocampal atrophy<br>- EEG: Mild diffuse encephalopathy
65	30s	Yes	Nontuberculous mycobacterial enecephalitis in SLE	- CSF profile: Normal<br>- CSF Bacterial culture: No growth<br>- CSF PCR for MTBC: Not detected<br>- CSF VDRL: Non-reactive<br>- CSF and Blood Autoimmune encephalitis antibodies panel: Negative<br>- CSF and Blood Paraneoplastic antibodies panel: Negative<br>- CSF and Blood Unclassified antibodies: Negative<br>- Oligoclonal bands: positive for intrathecal IgG synthesis<br>- CSF Cytospin: No cell<br>- MRI Brain with Gad: Bilateral striatal hyperSI T2/FLAIR more on right side<br>- MR Spetroscopy: Increased Cho peak while decreased Cr and NAA, non-specific pattern of neuronal loss and high cell membrane turnover, also presence of inverted lactate peak, representing anaerobic metabolism (Cho/Cr ratio = 3.9 and Cho/NAA ratio = 2.86). These MRS pattern can be observed in both inflammatory change and glioma<br>- Brain biopsy PCR: positive for *Mycobacterium celatum*
66	70s	Yes	Septic encephalopathy	- CSF profile: Normal<br>- CSF Bacterial culture: No growth<br>- CSF ME panel: Not detected<br>- CSF and Blood Autoimmune encephalitis antibodies panel: Negative<br>- CSF and Blood Paraneoplastic antibodies panel: Negative<br>- CSF and Blood Unclassified antibodies: Negative<br>- MRI Brain with Gad: Normal
67	30s	No	Epilepsy	- CSF profile: Normal<br>- CSF Bacterial culture: No growth<br>- CSF and Blood Unclassified antibodies: Negative<br>- MRI Brain with Gad: Normal<br>- EEG: Increased epileptogenicity over right frontocentral region
68	10s	No	Acute psychosis	- CSF profile: Normal<br>- CSF Bacterial culture: No growth<br>- CSF ME panel: Not detected<br>- CSF and Blood Autoimmune encephalitis antibodies panel: Negative<br>- CSF and Blood Unclassified antibodies: Negative<br>- MRI Brain with Gad: Normal

69	80s	No	Metabolic encephalopathy	- CSF profile: Normal<br>- CSF Bacterial culture: No growth<br>- CSF ME panel: Not detected<br>- CT Brain non contrast: Normal
70	50s	Yes	FTD-PSP	- CSF profile: Normal<br>- CSF and Blood Autoimmune encephalitis antibodies panel: Negative<br>- MRI Brain with Gad: Markedly atrophic of brainstem and bilateral cerebellum and middle cerebellar peduncles with hot cross bun sign in pons, probably MSA-C or SCA with brain atrophy with frontal predilection, more advance than age
71	80s	No	Epilepsy	- CSF profile: Normal<br>- CSF Bacterial culture: No growth<br>- CSF ME panel: Not detected<br>- CSF and Blood Autoimmune encephalitis antibodies panel: Negative<br>- CSF and Blood Paraneoplastic antibodies panel: Negative<br>- CSF and Blood Unclassified antibodies: Negative<br>- MRI Brain: Ischemic white matter change with multiple old infarction and brain atrophy<br>- EEG: 1 episode of electroclinical seizure arising from right temporal region

Determining the CSF NFL cut-off value

The ability of CSF NFL to distinguish between structural and non-structural causes of AOC was evaluated in the present study, yielding a moderate AUC of 0.75 (95% CI 0.63-0.88). To identify the optimal cut-off value, various approaches were considered. One common method is to select the threshold that maximizes the sum of sensitivity and specificity (ie. highest Youden index). In our study, this value for CSF NFL was found to be 808.8 pg/mL, resulting in a Youden index of 0.40, corresponding to a sensitivity of 83.3% and specificity of 56.5%. Given the moderate AUC when CSF NFL is used in isolation, it is expected that it may not perform optimally in all diagnostic scenarios. When utilizing this threshold, CSF NFL exhibits high sensitivity but low specificity. This can potentially limit its ability to rule out structural causes of AOC when the test is negative. To further assess the performance of CSF NFL, we conducted a sensitivity analysis, employing alternative methods as outlined in **Supplementary Table 2**, with the results presented in **Supplementary Table 3** and **Supplementary Figure 1**. All methods confirmed our initial finding that CSF NFL alone demonstrates only moderate diagnostic performance.

In clinical chemistry, a common strategy for addressing biomarkers with moderate performance is to establish two cut-off values. Individuals with biomarker levels below the lower cut-off can be confidently classified as negative, while those with levels above the higher cut-off can be similarly classified as positive. Those falling within the intermediate range, between the two cut-off values, are considered inconclusive and may require additional testing. Typically, these cut-off values are chosen to achieve either 90% sensitivity and 90% specificity, or, depending on the context, 95% or 97.5% sensitivity and specificity. In our study, the cut-off values for CSF NFL, corresponding to 90% sensitivity and specificity, were determined to be 501.42 pg/mL and 5648.01 pg/mL, respectively. By applying these cut-offs, we can accurately classify up to 49.3% of the participants (see **Supplementary Figure 2**). However, for the remaining 50.7% of participants, discrimination based solely on CSF NFL levels may not be conclusive. Additional testing or the incorporation of other relevant data into diagnostic models, as demonstrated in the latter part of this study, may be necessary.

Supplementary Table 2 Methods for Determining Optimal Cut-off Values

**Methods**	**Description**
Youden index	This method determines the cut-off value that maximizes the sum of sensitivity and specificity, resulting in the lowest mean error rates for both positive and negative results. This method is independent of prevalence.
Sens= spec	This method identifies the optimal cut-off value where sensitivity equals specificity. At this cut-off, positive and negative results are equally likely to be incorrect.
Cohen Kappa	This method determines the cut-off value that yields the maximum value of Cohen's Kappa in the contingency table.
Accuracy	This method determines the cut-off value that maximizes accuracy, representing the percentage of correctly classified results.
ROC (0,1)	This method determines the cut-off value by minimizing the distance between the ROC plot and the upper-left corner of the unit square.
95th percentile	This method sets the cut-off value at the 95th percentile of controls, which approximates the value 2 standard deviations above the mean in a normally distributed population.

Supplementary Table 3 Evaluating Diagnostic Parameters Across Various CSF NFL Thresholds in All 71 Participants.

**Diagnostic parameters**	**Methods used to determine the optimal cut-off values**					
	**Youden index**	**Sens= spec**	**Cohen Kappa**	**Accuracy**	**ROC (0,1)**	**95th percentile**
CSF NFL cut-offs, pg/mL	808.82	2253.04	808.82	268.93	2428.25	7669.42
Youden index	0.40	0.30	0.40	0.33	0.38	0.2
Sensitivity, %	83.33	64.58	83.33	97.92	64.58	29.17
Specificity, %	56.52	65.22	56.52	34.78	73.91	91.3
Accuracy, %	74.65	64.79	74.65	77.46	67.61	49.3
Positive predictive value, %	80.00	79.49	80.00	75.81	83.78	87.5
Negative predictive value, %	61.9	46.88	61.9	88.89	50.00	38.18
Positive likelihood ratio	1.92	1.86	1.92	1.50	2.48	3.35
Negative likelihood ratio	0.29	0.54	0.29	0.06	0.48	0.78

Supplementary Figure 1 Diagnostic performance of CSF NFL using different cut-off values. The varying sensitivities and specificities were plot against the cut-off applied (A). The coloured vertical lines are optimal cut-off values as suggested by different methods shown in **Table I**. Box-and-whisker plots, scatter plot as well as density plots of CSF NFL levels stratified by diagnostic groups were shown relative to the suggested cut-off values (B).

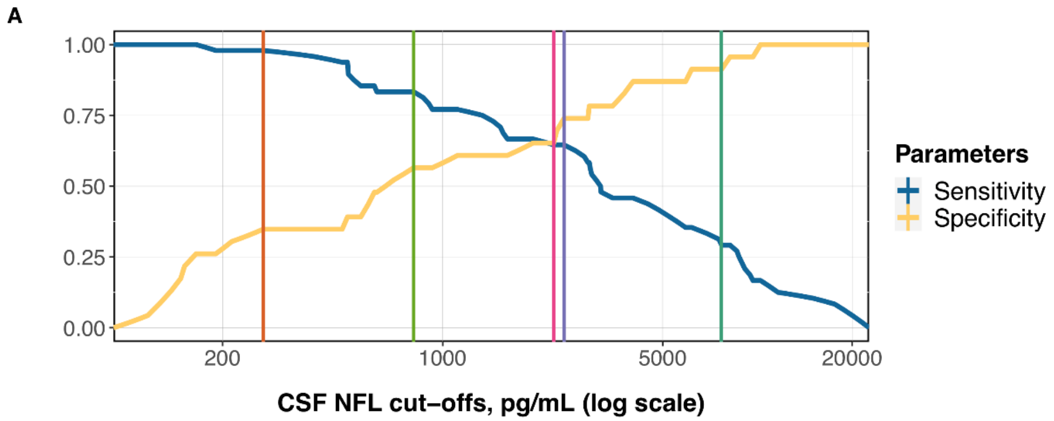

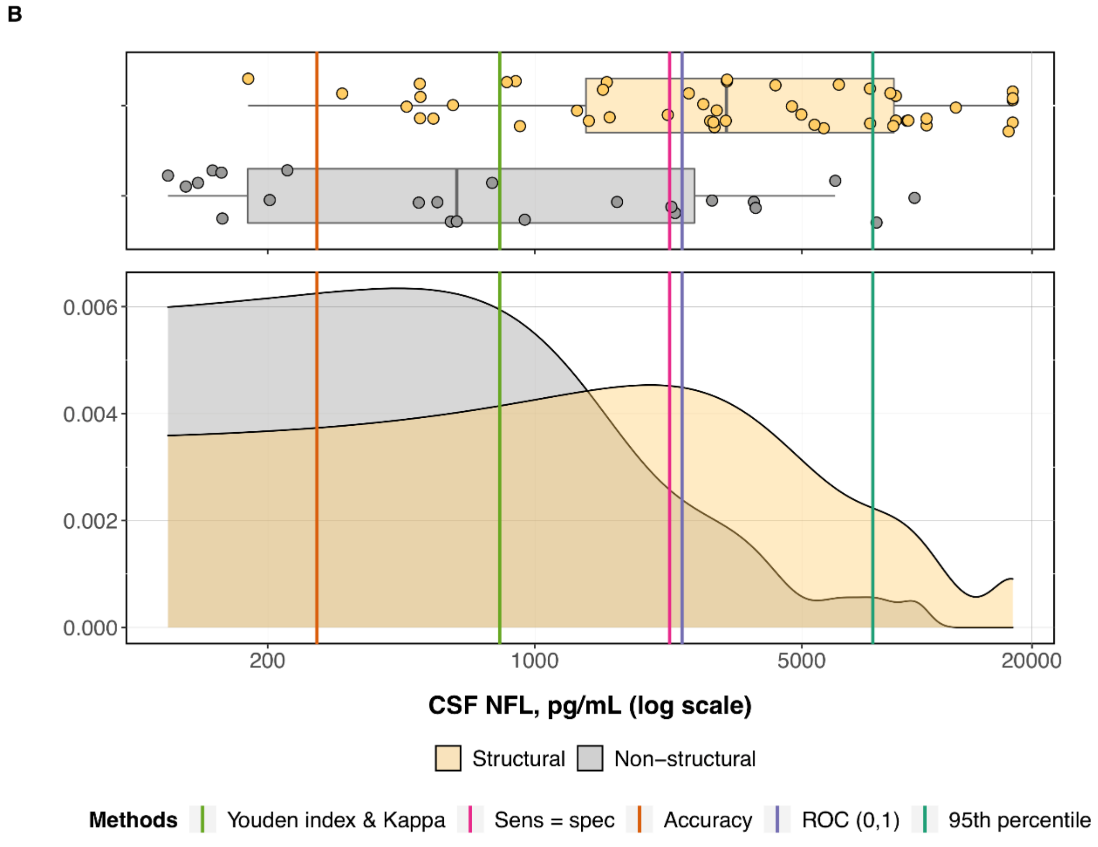

Supplementary Figure 2 Applying low and high cut-off values for CSF NFL using the 90% sensitivity and the 90% specificity cut-off values. The corresponding NFL levels were 501.42 and 5648.01 pg/mL, respectively. Using this approach, up to 49.7% of the participant can be classified using CSF NFL alone while the other 50.7% stays the *intermediate zone*.

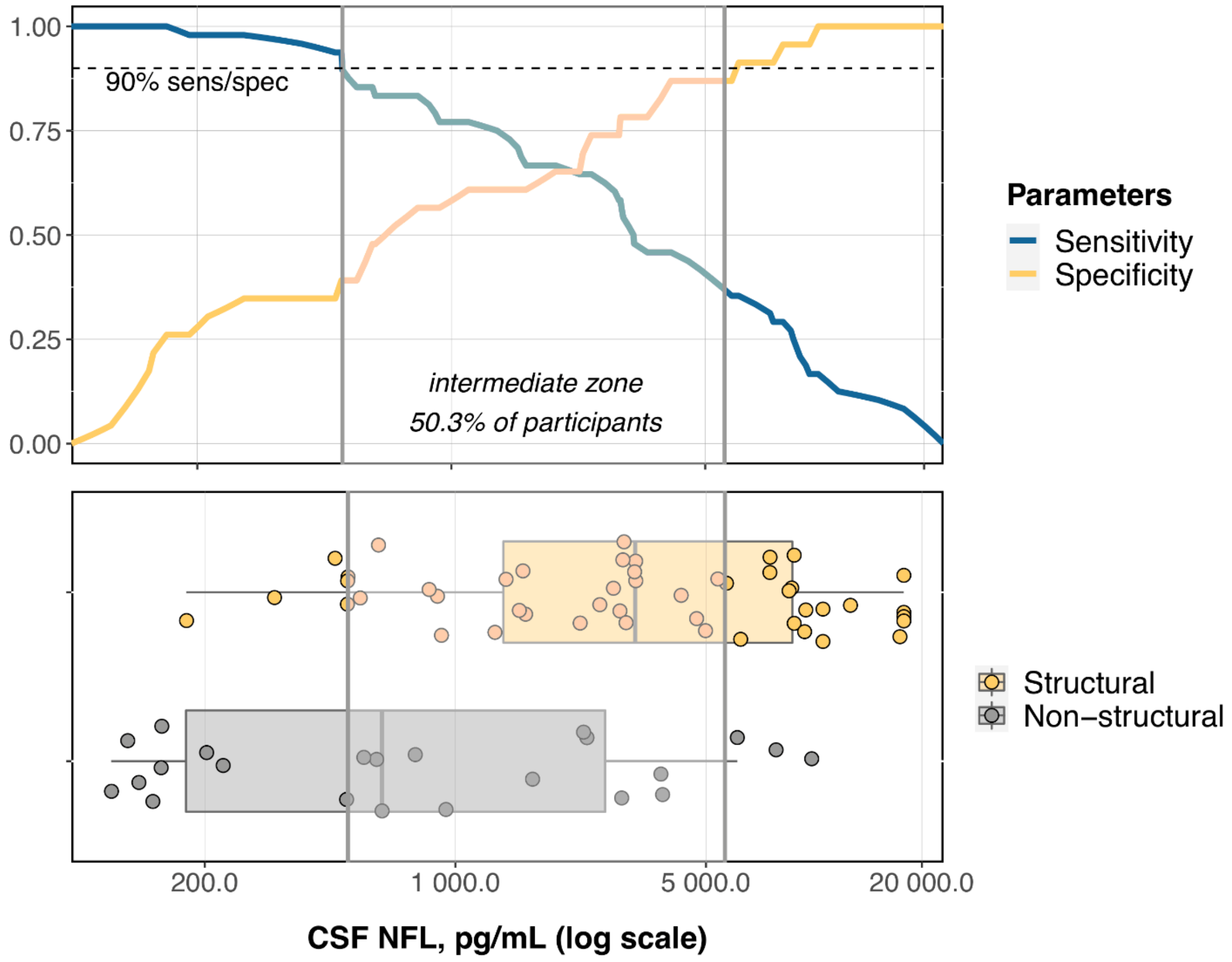

Logistic regression model

- Model

$$\log\left(\frac{\hat{y}^{(i)}}{1-\hat{y}^{(i)}}\right) = \text{logit}\left(\hat{y}^{(i)}\right) = f(x^{(i)}) = b_0 + b_1 x_1^{(i)} + \cdots + b_n x_n^{(i)}$$

$$\hat{y}^{(i)} = \frac{e^{b_0 + b_1 x_1^{(i)} + \cdots + b_n x_n^{(i)}}}{1 + e^{b_0 + b_1 x_1^{(i)} + \cdots + b_n x_n^{(i)}}}$$

where $\hat{y}^{(i)}$ *is the probability of a structural final diagnosis*

b_0 *is a constant*

b_n *are the coefficients of independent variables*

$x_n^{(i)}$ *are the independent variables*

Production model		coefficients	Feature Mean	Feature SD
**intercept**		**-0.0012**		
CSF NFL, pg/mL	x_1	-0.0015	7.58767545	1.452298694
Days after onset, days	x_2	-0.0013	2.538333648	1.498762928
CSF profile (1=normal,2=abnormal)	x_3	-0.0012	1.281690141	0.453024711
CSF protein, mg/dL	x_4	-0.0013	3.83802335	0.784341315
CSF WBC, cells/mm^3	x_5	-0.0011	1.808287812	1.290354562
Abnormal neuroexam (1=no,2=yes)	x_6	-0.0008	1.295774648	0.45963856

there are 6 parameters that serves as 6 independent variables: $x_1 - x_6$

and $b_0 = -0.0012$ *which the probability of a structural final diagnosis is as follows*

$$\hat{y}^{(i)} = \frac{e^{(-0.0012)+(-0.0015)x_1^{(i)}+(-0.0013)x_2^{(i)}+(-0.0012)x_3^{(i)}+(-0.0013)x_4^{(i)}+(-0.0011)x_5^{(i)}+(-0.008)x_6^{(i)}}}{1+e^{(-0.0012)+(-0.0015)x_1^{(i)}+(-0.0013)x_2^{(i)}+(-0.0012)x_3^{(i)}+(-0.0013)x_4^{(i)}+(-0.0011)x_5^{(i)}+(-0.008)x_6^{(i)}}}$$

$$\hat{y}^{(i)} = \frac{e^{(-0.0012)-(0.0015)x_1^{(i)}-(0.0013)x_2^{(i)}-(0.0012)x_3^{(i)}-(0.0013)x_4^{(i)}-(0.0011)x_5^{(i)}+(-0.008)x_6^{(i)}}}{1+e^{(-0.0012)-(0.0015)x_1^{(i)}-(0.0013)x_2^{(i)}-(0.0012)x_3^{(i)}-(0.0013)x_4^{(i)}-(0.0011)x_5^{(i)}+(-0.008)x_6^{(i)}}}$$

Prior to the input of the variables, standardization of the raw data has to be performed with z-score normalization. This can be achieved by using the mean and the SD shown in the tables above:

$$x' = \frac{x - \bar{x}}{\sigma}$$

$$x_n^{(i)} = \frac{x_{n(raw)} - \overline{x_n}}{x_{n_{SD}}}$$

where $x_{n(raw)}$ *is the raw data:* x_n

$\overline{x_n}$ *is the* Feature Mean

$x_{n_{SD}}$ *is the* Feature SD

Substituting $x_n^{(i)}$ the equation for *probability of a structural final diagnosis* estimating the is as follows:

$$\hat{y}^{(i)} = \frac{e^{[(-0.0012)-(0.0015)\left(\frac{x_1 - 7.58767544964565}{1.45229869420967}\right)-(0.0013)\left(\frac{x_2 - 2.53833364812671}{1.49876292811185}\right)-(0.0012)\left(\frac{x_3 - 1.28169014084507}{0.453024710507031}\right)-(0.0013)\left(\frac{x_4 - 3.83802334967106}{0.784341314580173}\right)-(0.0011)\left(\frac{x_5 - 1.80828781215944}{1.29035456172889}\right)-(0.008)\left(\frac{x_6 - 1.295774648}{0.459638559776921}\right)]}}{1 + e^{[(-0.0012)-(0.0015)\left(\frac{x_1 - 7.58767544964565}{1.45229869420967}\right)-(0.0013)\left(\frac{x_2 - 2.53833364812671}{1.49876292811185}\right)-(0.0012)\left(\frac{x_3 - 1.28169014084507}{0.453024710507031}\right)-(0.0013)\left(\frac{x_4 - 3.83802334967106}{0.784341314580173}\right)-(0.0011)\left(\frac{x_5 - 1.80828781215944}{1.29035456172889}\right)-(0.008)\left(\frac{x_6 - 1.295774648}{0.459638559776921}\right)]}}$$

```
a=(Math.expm1 ((-0.0012)-((0.0015)*((x1-mean1)/sd1))-((0.0013)*((x2-mean2)/sd2))-((0.0012)*((x3-mean3)/sd3))-((0.0013)*((x4-mean4)/sd4))-((0.0011)*((x5-mean5)/sd5))+((0.0008)*((x6-mean6)/sd6))))/(1+(Math.expm1 ((-0.0012)-((0.0015)*((x1-mean1)/sd1))-((0.0013)*((x2-mean2)/sd2))-((0.0012)*((x3-mean3)/sd3))-((0.0013)*((x4-mean4)/sd4))-((0.0011)*((x5-mean5)/sd5))-((0.0008)*((x6-mean6)/sd6)))));
```

Section & Topic	No	Item	Reported on page #
**TITLE OR ABSTRACT**			
	**1**	Identification as a study of diagnostic accuracy using at least one measure of accuracy (such as sensitivity, specificity, predictive values, or AUC)	3
**ABSTRACT**			
	**2**	Structured summary of study design, methods, results, and conclusions (for specific guidance, see STARD for Abstracts)	3
**INTRODUCTION**			
	**3**	Scientific and clinical background, including the intended use and clinical role of the index test	5-6
	**4**	Study objectives and hypotheses	6
**METHODS**			
Study design	**5**	Whether data collection was planned before the index test and reference standard were performed (prospective study) or after (retrospective study)	6
Participants	**6**	Eligibility criteria	6-7
	**7**	On what basis potentially eligible participants were identified (such as symptoms, results from previous tests, inclusion in registry)	6-7
	**8**	Where and when potentially eligible participants were identified (setting, location and dates)	6
	**9**	Whether participants formed a consecutive, random or convenience series	7
Test methods	**10a**	Index test, in sufficient detail to allow replication	8
	**10b**	Reference standard, in sufficient detail to allow replication	8-9
	**11**	Rationale for choosing the reference standard (if alternatives exist)	8
	**12a**	Definition of and rationale for test positivity cut-offs or result categories of the index test, distinguishing pre-specified from exploratory	9
	**12b**	Definition of and rationale for test positivity cut-offs or result categories of the reference standard, distinguishing pre-specified from exploratory	9
	**13a**	Whether clinical information and reference standard results were available to the performers/readers of the index test	8
	**13b**	Whether clinical information and index test results were available to the assessors of the reference standard	8
Analysis	**14**	Methods for estimating or comparing measures of diagnostic accuracy	9-10
	**15**	How indeterminate index test or reference standard results were handled	8
	**16**	How missing data on the index test and reference standard were handled	9
	**17**	Any analyses of variability in diagnostic accuracy, distinguishing pre-specified from exploratory	9
	**18**	Intended sample size and how it was determined	7
**RESULTS**			
Participants	**19**	Flow of participants, using a diagram	-
	**20**	Baseline demographic and clinical characteristics of participants	12-13
	**21a**	Distribution of severity of disease in those with the target condition	12-13
	**21b**	Distribution of alternative diagnoses in those without the target condition	14
	**22**	Time interval and any clinical interventions between index test and reference standard	Supplementary material
Test results	**23**	Cross tabulation of the index test results (or their distribution) by the results of the reference standard	Supplementary material
	**24**	Estimates of diagnostic accuracy and their precision (such as 95% confidence intervals)	14
	**25**	Any adverse events from performing the index test or the reference standard	-
**DISCUSSION**			
	**26**	Study limitations, including sources of potential bias, statistical uncertainty, and generalisability	18-19
	**27**	Implications for practice, including the intended use and clinical role of the index test	19
**OTHER INFORMATION**			

cc BY SA

	**28**	Registration number and name of registry	7
	**29**	Where the full study protocol can be accessed	7
	**30**	Sources of funding and other support; role of funders	20

STARD 2015

AIM

STARD stands for “Standards for Reporting Diagnostic accuracy studies”. This list of items was developed to contribute to the completeness and transparency of reporting of diagnostic accuracy studies. Authors can use the list to write informative study reports. Editors and peer-reviewers can use it to evaluate whether the information has been included in manuscripts submitted for publication.

EXPLANATION

A **diagnostic accuracy study** evaluates the ability of one or more medical tests to correctly classify study participants as having a **target condition.** This can be a disease, a disease stage, response or benefit from therapy, or an event or condition in the future. A medical test can be an imaging procedure, a laboratory test, elements from history and physical examination, a combination of these, or any other method for collecting information about the current health status of a patient.

The test whose accuracy is evaluated is called **index test.** A study can evaluate the accuracy of one or more index tests. Evaluating the ability of a medical test to correctly classify patients is typically done by comparing the distribution of the index test results with those of the **reference standard**. The reference standard is the best available method for establishing the presence or absence of the target condition. An accuracy study can rely on one or more reference standards.

If test results are categorized as either positive or negative, the cross tabulation of the index test results against those of the reference standard can be used to estimate the **sensitivity** of the index test (the proportion of participants *with* the target condition who have a positive index test), and its **specificity** (the proportion *without* the target condition who have a negative index test). From this cross tabulation (sometimes referred to as the contingency or “2x2” table), several other accuracy statistics can be estimated, such as the positive and negative **predictive values** of the test. Confidence intervals around estimates of accuracy can then be calculated to quantify the statistical **precision** of the measurements.

If the index test results can take more than two values, categorization of test results as positive or negative requires a **test positivity cut-off**. When multiple such cut-offs can be defined, authors can report a receiver operating characteristic (ROC) curve which graphically represents the combination of sensitivity and specificity for each possible test positivity cut-off. The **area under the ROC curve** informs in a single numerical value about the overall diagnostic accuracy of the index test.

The **intended use** of a medical test can be diagnosis, screening, staging, monitoring, surveillance, prediction or prognosis. The **clinical role** of a test explains its position relative to existing tests in the clinical pathway. A replacement test, for example, replaces an existing test. A triage test is used before an existing test; an add-on test is used after an existing test.

Besides diagnostic accuracy, several other outcomes and statistics may be relevant in the evaluation of medical tests. Medical tests can also be used to classify patients for purposes other than diagnosis, such as staging or prognosis. The STARD list was not explicitly developed for these other outcomes, statistics, and study types, although most STARD items would still apply.

DEVELOPMENT

This STARD list was released in 2015. The 30 items were identified by an international expert group of methodologists, researchers, and editors. The guiding principle in the development of STARD was to select items that, when reported, would help readers to judge the potential for bias in the study, to appraise the applicability of the study findings and the validity of conclusions and recommendations. The list represents an update of the first version, which was published in 2003.

More information can be found on [http://www.equator-network.org/reporting-guidelines/stard.](http://www.equator-network.org/reporting-guidelines/stard)
